# Occupational Stress and Burnout Among Public Health Professionals During the COVID-19 Pandemic in Morocco

**DOI:** 10.3390/healthcare13212700

**Published:** 2025-10-26

**Authors:** Amal Amellah, Aziza Menouni, Kaoutar Chbihi, Hala Chetouani, Said Abou-Said, Tarik Abchouch, Lode Godderis, Samir El Jaafari, Mohammed Amane

**Affiliations:** 1 Human Epidemiology and Environmental Health Team, Faculty of Sciences, Moulay Ismail University, Meknes 50000, Moroccochetouanihala@gmail.com (H.C.); saidabousaid06@gmail.com (S.A.-S.); abchouch.tarik@gmail.com (T.A.); s.eljaafari@gmail.com (S.E.J.); amanemek@hotmail.fr (M.A.); 2 Environment & Health Unit, Department of Public Health & Primary Care, Faculty of Medicine, Katholieke Universiteit Leuven, 3000 Leuven, Belgium; aziza.menouni@kuleuven.be (A.M.); lode.godderis@kuleuven.be (L.G.)

**Keywords:** COVID-19, healthcare professionals, stress, burnout

## Abstract

Background: The COVID-19 pandemic posed a severe psychological burden on healthcare professionals worldwide, yet little evidence exists from North African low- and middle-income countries. Objective: This study assessed levels of perceived stress and burnout among Moroccan healthcare workers and explored associated occupational risk factors, based on retrospective data collected in 2022. Methods: A cross-sectional survey was conducted among 200 physicians and nurses (*n* = 200) from five public hospitals in Meknes. Validated instruments, the Perceived Stress Scale (PSS-14) and the Maslach Burnout Inventory (MBI-HSS), were used. Descriptive statistics, chi-square tests with Cramér’s V, and ordinal logistic regression analyses were performed using IBM SPSS Statistics version 26. Results: High proportions of healthcare professionals reported elevated stress and clinical burnout. Occupational risk factors such as working in intensive care or COVID-19 units, on-call shift schedules, and sleep disturbances were strongly associated with adverse outcomes. Conclusions: Moroccan healthcare professionals experienced persistent psychological strain during and after the pandemic. The novelty of this study lies in documenting post-pandemic data from 2022 in a North African setting, providing timely evidence of long-term impacts. These findings underscore the urgent need for organizational reforms and targeted psychological support programs to protect the mental health of healthcare workers in future health crises.

## 1. Introduction

As a major health crisis, the COVID-19 pandemic has affected the global health system, especially health professionals, who were the most exposed to the outbreak, affecting not only their physical health but also their mental and psychological well-being [1].

In Morocco, this pandemic unfolded within a health system challenged by chronic workforce shortages and regional inequalities. According to the 2023 health map published by the Ministry of Health and Social Protection, approximately 30,643 physicians (public and private) were practicing about 1 per 1255 inhabitants alongside 38,725 paramedical staff in the public sector [2]. By late 2022, Morocco had recorded over 1.26 million confirmed COVID-19 cases and nearly 16,300 deaths [3]. The pandemic response necessitated substantial mobilization: the nationwide vaccination campaign (launched January 2021) engaged 25,631 frontline health workers operating across 3047 fixed and more than 10,000 mobile vaccination sites [4]. Against this backdrop of constrained workforce capacity and unprecedented mobilization, frontline healthcare workers operated under sustained pressure, translating into demanding working conditions and heightened psychosocial risk. Specifically, extended shifts, emotional exhaustion, a lack of protective gear, and the fear of contaminating themselves and their family were all challenges faced by healthcare workers [5]. These pressures, which were frequently accompanied by social isolation and the psychological toll of confronting high death rates, further aggravated long-term mental health issues [6].

The psychological concerns mostly recorded among this population are stress and burnout, which are closely related and worsened during the pandemic [7]. Stress is generally defined as the body’s response to external or internal demands [8]. Whereas perceived stress a concept developed by Cohen et al., refers to the subjective appraisal of these demands as exceeding one’s coping capacity [9]. Burnout, on the other hand as defined by the world health organization, is a work-related syndrome characterized by emotional exhaustion, depersonalization, and reduced personal accomplishment [10]. Burnout has been linked to a variety of concerns, including higher rates of absenteeism, medical errors, and lower quality patient care. As a result, it has gained recognition as a major public health and institutional concern [11].

Across the globe, the rate of major depression escalated by approximately 27.6% in 2020, a consequence of the repercussions of the pandemic. This has led to the emergence of nearly 53 million new cases, with women experiencing a disproportionate impact [12].

Multiple studies from high-income nations have demonstrated the psychosocial impact of the pandemic on health care professionals [13]. Yet, most of these investigations were carried out during the early surge of COVID-19, capturing only the acute psychological responses to the crisis. In contrast, our study is distinguished by its focus on the post-peak phase: data were collected in 2022, a period when healthcare professionals had already endured the peak of the pandemic and could reflect on both immediate and persistent consequences. This timing provides a novel perspective, enabling the assessment of enduring stress and burnout beyond the initial emergency. Several studies have investigated the psychological burden of the COVID-19 pandemic on healthcare workers in diverse settings. In Asia and Europe structured psychosocial programs, tele-consultation services, and organizational measures were introduced to mitigate stress and burnout among frontline staff [14,15]. In the United States, outbreaks among healthcare personnel persisted even in highly vaccinated settings, underscoring their ongoing vulnerability and the need for long-term occupational health policies [16]. Furthermore, a recent global synthesis emphasized that the mental health impact of the pandemic extends beyond the acute crisis, requiring sustained preventive strategies and structural reforms [17]. However, limited data exist from countries with low or middle incomes, particularly those in North Africa [18]. In Morocco, few studies have examined the psychological burden of the COVID-19 pandemic on healthcare professionals, yet the available evidence consistently points to a high prevalence of distress. An investigation in Agadir reported a substantial risk of burnout among hospital staff during the pandemic, highlighting the impact of workload and occupational conditions [19]. Similarly, a study in southern Morocco found a significant deterioration in quality of life among healthcare workers, strongly linked to stress and anxiety during the crisis [20]. By situating our work in Morocco, we respond to this geographical gap while also advancing knowledge on the longer-term psychosocial effects of COVID-19, thereby highlighting the need for retrospective research to capture its persistent effects of COVID-19 on mental health and to inform sustainable preventive strategies.

The purpose of this study is to investigate the prevalence and predictors of perceived stress and burnout among healthcare professionals in the city of Meknes in Morocco, using data collected in 2022. We hypothesize that healthcare professionals involved in patient care during the COVID-19 pandemic are likely to present a high prevalence of stress and burnout, and that the pandemic context has further amplified these occupational mental health challenges.

## 2. Materials and Methods

### 2.1. Study Design and Setting

Our study used a cross-sectional and descriptive design and was undertaken in five public hospitals in Morocco’s Meknes prefecture from March to June 2022. These facilities are the region’s principal clinical care institutions, providing both medical and surgical treatments, including units dedicated to COVID-19 care.

Meknes is a major city located in the Fez-Meknes region of northern Morocco with around 568,000 inhabitants. Meknes has a wide range of healthcare facilities, from primary care centers to regional and military hospitals, which played a central role in managing severe COVID-19 cases, particularly during the Delta wave in 2021. Furthermore, as a geographic and economic crossroad, Meknes experienced a sustained strain on its health system throughout the pandemic, partly due to its strategic location and population density. It offers a unique setting to capture both urban and regional (surrounding rural areas, commuting flows) healthcare realities, allowing a broader understanding of healthcare professionals’ experiences in the post-pandemic period.

### 2.2. Sampling Procedure and Eligibility Criteria

The study focused on the healthcare professionals, essentially doctors and nurses, who operated at the selected hospitals during the study period. A stratified random sampling strategy was used to ensure representativeness across hospitals. The sampling frame consisted of official staff lists of physicians and nurses provided by the administration of each hospital. Each hospital constituted a stratum, and the number of participants selected from each stratum was proportional to the size of its staff. Within each hospital, participants were randomly drawn using a random number generator applied to the staff lists. Both physicians and nurses were eligible, and proportional representation was preserved to reflect the actual distribution of professionals across hospitals.

We calculated the Sample size OpenEpi (v3) using Cochran’s single-proportion formula.

n = [DEFF × Np(1 − p)]/[(d2/Z21 − α/2 × (N − 1) + p × (1 − p)]

where n (required sample size), DEFF (design effect, simple random sampling) = 1.0, N (finite population size) = 806, p (anticipated proportion, maximum variance) = 0.50, d (absolute precision, ±5 percentage points) = 0.05, Z1 − α/2Z_{1 − α/2}Z1 − α/2 (two-sided standard normal quantile for 90% confidence) = 1.645.

We applied a confidence level of 90% (Z = 1.645) to ensure an acceptable balance between statistical rigor and the feasibility of field data collection under constrained resources. In the calculation, p was fixed at 0.5, which is commonly adopted in sample size determination as it maximizes variance and ensures the largest, most conservative estimate. The source population was defined as all physicians and nurses working in the five public hospitals of Meknes (N = 806), based on administrative human resources records.

A total of 204 healthcare professionals were initially invited to participate across five public hospitals in Meknes. Of these, 202 returned complete questionnaires, resulting in an overall response rate of 99.0%. The distribution of participants was as follows: Hospital 1 (*n* = 77, 76 completed, 98.7%), Hospital 2 (*n* = 51, 51 completed, 100%), Hospital 3 (*n* = 31, 31 completed, 100%), Hospital 4 (*n* = 34, 34 completed, 100%), and Hospital 5 (*n* = 11, 10 completed, 90.9%). Within this sample, 54 were physicians and 150 were nurses. Non-response was therefore minimal (two incomplete questionnaires) and did not appear to be systematically associated with any particular hospital or professional group. A potential selection bias remains, and the findings mainly apply to frontline physicians and nurses in Meknes public hospitals.

The study included medical physicians and nurses, who held a tenured or contractual position at a hospital or clinic in Morocco and had worked during the COVID-19 period. Eligible participants were required to have been regularly active in clinical practice and to have provided informed consent prior to data collection. Exclusion criteria were as follows: non-clinical or administrative staff, medical residents, individuals currently under psychotropic or antidepressant medication, and those whose questionnaires were incomplete. Although these exclusions may slightly affect representativeness, they were necessary to ensure the internal validity of the study. All statistical analyses were performed using IBM SPSS Statistics version 26.0 (IBM Corp., Armonk, NY, USA). Healthcare professionals were approached directly at their workplace after obtaining administrative authorization. An information sheet was provided to explain the study, and participation was voluntary. Confidentiality and anonymity of the data were carefully safeguarded throughout both the collection and the analysis phases.

### 2.3. Data Collection Tools

Data was gathered using a structured questionnaire, completed independently by respondents, and composed of three distinct sections: (1) sociodemographic and occupational data, (2) the Perceived Stress Scale (PSS-14), and (3) the Maslach Burnout Inventory (MBI).

Perceived stress was assessed using the 14-item Perceived Stress Scale (PSS-14). The instrument evaluates the frequency of stress-related thoughts and feelings over the previous month on a 5-point Likert scale, with higher scores reflecting greater perceived stress [21]. It proved strong reliability and validity across several demographics, including health care professionals, and has been translated and validated in multiple languages [22,23]. we used validated Arabic and French versions of the PSS, including the Arabic version validated by Almadi et al. which demonstrated good internal consistency (Cronbach’s α ≈ 0.80) and test–retest reliability (ICC ≈ 0.90) [24]. In our study, we opted for the PSS-14 rather than the abbreviated 10-item version in order to capture a broader range of perceived stress dimensions (unpredictability, uncontrollability, overload). This choice ensured a wider scoring range and a balanced distribution of positively and negatively worded items, thereby limiting wording bias. According to standard cutoffs reported in the literature, total PSS-14 scores were categorized as follows: low stress (0–18), moderate stress (19–37), and high stress (38–56).

Burnout was assessed using the Maslach Burnout Inventory (MBI), developed by Christina Maslach and Susan E. Jackson [25], which is considered a reliable tool to assess burnout across three dimensions and is widely used in healthcare research [26]. Burnout was assessed using the French version of the Maslach Burnout Inventory–Human Services Survey (MBI-HSS), previously validated in francophone populations and widely used among healthcare professionals [27]. It comprises 22 questions that evaluate work-related feelings. Every question is scored on a 7-point Likert scale that ranges from 0 (never) to 6 (every day), indicating how often respondents experience these emotions in their professional context [28]. Higher emotional exhaustion and depersonalization scores, along with lower personal accomplishment levels, were linked to an increased risk of burnout [29]. Following commonly applied cut-off points in the literature, Emotional Exhaustion (EE) was categorized as low (≤16), moderate (17–26), or high (≥27). Depersonalization (DP) was classified as low (≤6), moderate (7–12), or high (≥13). Personal Accomplishment (PA) was scored inversely, where low accomplishment was defined as ≤31, moderate as 32–38, and high as ≥39. Based on an existing classification approach used in previous research, participants were categorized into three levels of burnout severity: burnout syndrome (high Personal Accomplishment Emotional Exhaustion or Depersonalization, or low Personal Accomplishment), intermediate burnout (moderate level in at least one dimension), and no burnout (low Emotional Exhaustion and Depersonalization, and high Personal Accomplishment) [30]. In our sample, internal consistency was satisfactory, with Cronbach’s alpha of 0.85 for the PSS-14, and 0.89, 0.81, and 0.78 for emotional exhaustion, depersonalization, and personal accomplishment subscales, respectively.

Sociodemographic and occupational covariates were considered as potential predictors. Sex was recorded as female or male. Marital status was classified as single or married. Occupational variables included profession (nurse or physician), hospital department (medical, surgical, intensive care unit, or COVID-19 unit), and work schedule, which was defined as either on-call (including night and weekend duties) or administrative (fixed daytime schedule). Additional work-related factors comprised assignment to COVID-19 units (yes or no) and self-reported sleep disturbance (yes or no). All covariates were treated as categorical variables and entered as independent variables in the regression analyses

To improve participation rates, the questionnaire was administered in Arabic and French, using two formats: a paper-based format distributed among healthcare workers in nearby hospitals who provided prior administrative authorization, and an online version created using Google Forms (Google LLC, Mountain View, CA, USA; version accessed in March 2022). and randomly sent to 104 hospital medical staff in remote areas. The questions were designed based on similar studies conducted in other countries, and were peer-reviewed by a governmental physician from the Department of Epidemiology. All data was manually entered into SPSS after being carefully verified, to ensure accuracy and consistency. Missing data were handled using listwise deletion: questionnaires with ≥10% missing responses or missing outcome variables were excluded, resulting in 200 complete cases analyzed out of 204 initially targeted.

### 2.4. Data Processing and Analysis

The data was coded, cleaned, and analyzed using IBM SPSS Statistics version 26 (IBM Corp., Armonk, NY, USA). Descriptive analyses first summarized participants’ sociodemographic and occupational characteristics, as well as their levels of perceived stress and burnout. Categorical variables appear as percentages and frequencies. Bivariate analyses were performed to assess associations between sociodemographic factors and levels of perceived stress and burnout. Chi-square tests were used to evaluate statistical significance, and Cramér’s V was calculated to measure the strength of association. Cramér’s V values were interpreted as follows: 0–0.1 = negligible; 0.1–0.3 = weak; 0.3–0.5 = moderate; >0.5 = strong association. Ordinal logistic regression analyses were then conducted to identify independent predictors of perceived stress and emotional exhaustion, both treated as ordinal outcome variables with three levels (low, moderate, high). Variables with *p* < 0.20 in bivariate analysis were considered for inclusion in the multivariable models. The proportional odds (parallel lines) assumption was assessed using the Test of Parallel Lines. Only models satisfying this assumption (*p* > 0.05) were retained. Multicollinearity was examined using variance inflation factors (VIF), with VIF > 5 considered indicative of collinearity. Model adequacy was further evaluated using Pearson and Deviance goodness-of-fit statistics, and explanatory power was assessed with pseudo R^2^ measures (Cox & Snell, Nagelkerke, McFadden). Although models were adjusted for relevant sociodemographic and occupational covariates, the possibility of residual confounding due to unmeasured variables (e.g., prior mental health conditions, social support) cannot be entirely excluded.

Adjusted odds ratios (aORs) and 95% confidence intervals (CIs) were reported to estimate the strength of association between predictors and outcomes. All tests were two-tailed, and a *p*-value < 0.05 was considered statistically significant.

### 2.5. Ethical Considerations

The Ethics Committee of Moulay Ismail University, Meknes, Morocco, approved the study on 1 April 2021 (Reference No: 02/21). All participants provided written informed consent. Data were anonymized and treated with strict confidentiality. The protocol and the questionnaire were peer-reviewed by colleagues from the Environment and Health Unit at the KU Leuven in Belgium.

## 3. Results

### 3.1. Descriptive Results

Table 1 summarizes the sociodemographic and professional characteristics of the participants. The majority were nurses, predominantly female, with more than half being married. Most participants reported on-call work schedules, and nearly two-thirds were assigned to COVID-19 units and experienced sleep disorders during the pandemic.

Table 2 presents the prevalence of perceived stress and burnout dimensions. More than half of the participants reported high stress and emotional exhaustion, while about one-third experienced high depersonalization. Low personal accomplishment was particularly frequent, indicating reduced professional fulfillment. Overall, burnout severity was substantial, with over half of respondents classified as having burnout syndrome, one-third as moderate burnout, and less than one-fifth showing no signs of burnout.

Table 3 presents the distribution of burnout severity, showing that half of the participants met the criteria for burnout syndrome, while only a minority reported no signs of burnout.

### 3.2. Bivariate Associations

Bivariate analyses were used to investigate the associations between sociodemographic and occupational factors, as well as perceived stress and burnout dimensions. Chi-square tests were applied to test the statistical associations between variables, and Cramér’s V was then calculated to estimate the strength of these associations.

**Figure 1:**
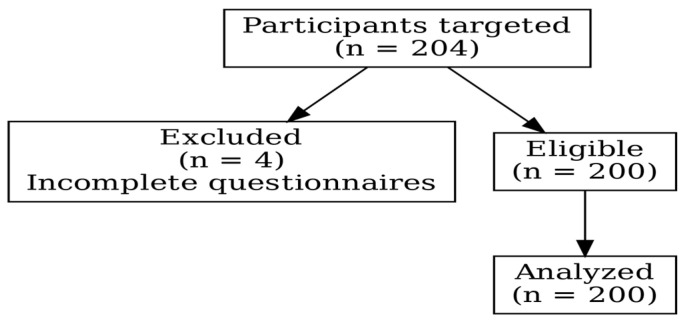
Flow diagram of participants according to STROBE guidelines.

Table 4 and Table 5 summarize the associations between sociodemographic and occupational characteristics and the levels of perceived stress and burnout dimensions. Figure 1 presents a heat map illustrating the strength of association using Cramer’s V value. The strength of associations, measured by Cramer’s V, is visualized in the heat map presented in Figure 1.

Table 4 shows significant associations between perceived stress and gender, occupation, work department, work schedule, COVID-19 assignment, and sleep disorders (*p* < 0.05). High stress was particularly common among females, nurses, ICU and COVID unit staff, those on on-call schedules, and participants with sleep disorders. No significant association was found with marital status.

Table 5 presents the associations between burnout dimensions and sociodemographic and occupational variables. Emotional exhaustion, depersonalization, and low personal accomplishment were significantly associated with occupation, department, work schedule, COVID-19 assignment, and sleep disorders (*p* < 0.05). Nurses, ICU staff, and professionals working under on-call schedules or assigned to COVID-19 units consistently showed higher levels of emotional exhaustion and depersonalization, along with lower personal accomplishment. In contrast, no significant associations were observed with gender or marital status.

**Figure 2:**
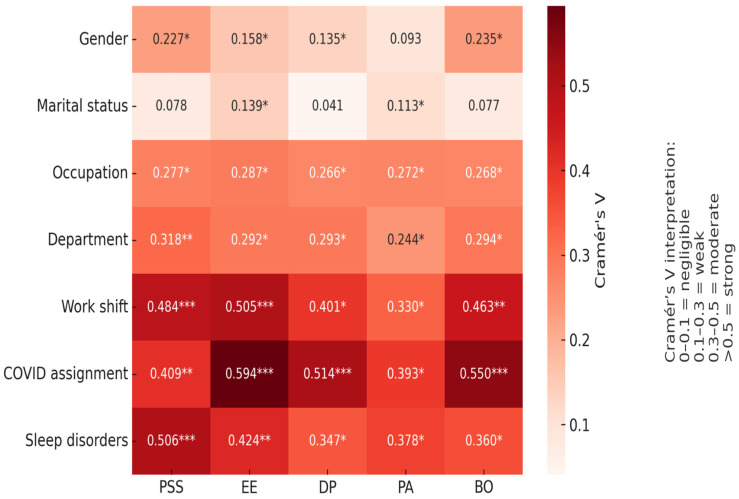
Visualization of associations (Cramér’s V) between socio-demographic variables and stress/burnout dimensions among health care professionals. PSS: Perceived stress scale, EE: Emotional Exhaustion, DP: Depersonalization, PA: Personal Accomplishment, BO: Burn out; *: Weak association, **: Moderate association ***: Strong association.

Figure 2 illustrates that workplace-related factors had the strongest associations with perceived stress and burnout among healthcare professionals. Sleep disorders and COVID-19 assignment emerged as the most influential determinants, followed by shift work, particularly in relation to emotional exhaustion and perceived stress. In contrast, sociodemographic variables such as gender and marital status showed only weak associations, suggesting that occupational conditions play a more decisive role in predicting stress and burnout.

### 3.3. Statistical Modelisation

Ordinal logistic regression was used to identify the independent predictors of perceived stress and emotional exhaustion. Emotional exhaustion was selected as one of the three dimensions of burnout for regression analysis, as it is considered the fundamental component of burnout and is most closely related to persistent work-related stress. The dependent variables, perceived stress and emotional exhaustion, were treated as ordinal outcomes with three levels: low, moderate, and high. Variables with a *p*-value of less than 0.20 in the bivariate analysis were included in the multivariable model. The Test of Parallel Lines was used to test the proportional odds assumption. Table 6 and Table 7 show the adjusted odds ratios (AORs) and 95% confidence intervals (CIs) for each predictor. COVID-19 unit assignment, work schedule, and sleep difficulties were identified as the most significant independent predictors of perceived stress and emotional exhaustion, respectively. A 90% confidence level (Z = 1.645) was used for the a priori sample size calculation to ensure feasibility in recruiting participants under post-pandemic field conditions. However, all subsequent statistical analyses were conducted with 95% confidence intervals, in line with international standards for epidemiological research and to facilitate comparability with previous studies

Table 6 shows the results of the ordinal logistic regression for perceived stress. Compared to ICU staff, healthcare professionals in the medical and COVID-19 departments were significantly less likely to report high stress levels, with an approximately 87% reduction in odds. Administrative schedules also appeared protective, reducing the likelihood of high stress compared to on-call systems. Similarly, those not assigned to COVID-19 units and those without sleep disturbances had significantly lower odds of high stress, underlining the critical role of sleep quality and work organization in mental health. In contrast, occupation (nurse vs. doctor) and working in surgical departments were not significant predictors after adjustment.

Table 7 presents the regression results for emotional exhaustion. Compared with ICU staff, healthcare professionals in medical and COVID-19 departments were about 76% less likely to report high levels of emotional exhaustion, suggesting that ICU work was particularly demanding during the pandemic. Administrative schedules also showed a protective effect, reducing the likelihood of emotional exhaustion compared with on-call work. Sleep disturbances showed a possible association with emotional exhaustion, although this did not reach statistical significance. No significant differences were observed between nurses and doctors, indicating that occupational role did not independently predict emotional exhaustion.

## 4. Discussion

This study aimed to investigate the prevalence and determinants of perceived stress and burnout among healthcare professionals in following the COVID-19 pandemic. According to our findings, over half of the participants reported high levels of perceived stress and emotional exhaustion. These rates indicate that the epidemic continued to exert a psychological impact on frontline workers even after it has passed.

Globally numerous studies conducted during the COVID-19 pandemic worldwide have documented a high prevalence of perceived stress and all three dimensions of burnout among healthcare professionals. In line with our findings, a nationwide, cross-sectional study including 20,947 healthcare workers in the United States found that 49.0% experienced burnout symptoms. Nursing staff were identified as one of the groups most affected [31]. Comparable proportions have been documented across Europe, with a study from the Czech Republic and Slovakia highlighting high levels of emotional exhaustion, depersonalization, and reduced personal accomplishment among hospital employees [32]. In the Asian context, the evidence from a study conducted in Thailand, including 517 hospital workers, reported that 41.97% experienced moderate to high perceived stress [33]. Similarly, a study conducted in Japan found that 38.0% of healthcare workers experienced high emotional exhaustion, 34.0% reported depersonalization, and 29% were affected by low personal accomplishment [34]. Across Arab MENA countries, studies have reported analogous findings, where studies consistently revealed high levels of stress and burnout among healthcare workers. A nine-country Eastern Mediterranean survey in 2020 underscored the pervasive regional burden [35]. Evidence from Tunisia, Lebanon, and Egypt further confirmed this trend, with prevalence rates ranging from moderate-to-high burnout in Lebanon to extremely severe psychological distress in Egypt and Tunisia [36,37,38]. In Morocco, research on healthcare professionals’ mental health remains scarce and largely outdated, with only a few studies specifically addressing the impact of COVID-19 and its associated risk factors [39]. For instance, studies conducted in Settat, northern Morocco, and the cities of Casablanca and Fez consistently revealed a high prevalence of burnout and psychological distress, with significant associations with gender, age, professional category, and frontline assignment [40,41,42]. These alarming rates can be explained by the intense pressure exerted by the COVID-19 pandemic, which forced healthcare professionals to work long hours without sufficient rest in response to the large influx of patients. The consistency of findings across different countries and healthcare systems suggests that the pandemic has had a universally disruptive impact on mental health, particularly among frontline staff.

When comparing professional roles, our findings revealed marked disparities between doctors and nurses in terms of perceived stress and burnout. Nurses reported significantly higher levels of perceived stress, emotional exhaustion, and low personal accomplishment than physicians, although differences in depersonalization were not statistically significant. These results align with previous studies from Mexico and Canada, both of which identified nurses as particularly vulnerable to occupational stress and burnout compared to physician [43,44]. In contrast, Ruiz Fernández et al. reported different patterns in Spain, where physicians displayed higher burnout and compassion fatigue, while nurses experienced greater compassion satisfaction and no significant differences in perceived stress [45]. Such discrepancies may reflect contextual differences across healthcare systems, levels of resource availability, or cultural factors shaping the professional experience during the pandemic. The greater vulnerability of nurses observed in our study is likely explained by the nature of their role, which entails more frequent and prolonged patient contact, heavier workloads, and reduced autonomy in clinical decision-making.

Furthermore, working in intensive care units (ICU) was strongly associated with higher stress and emotional exhaustion. It is inherently associated with elevated stress levels, even under normal circumstances [46]. Notably studies from Italy and China conducted during the COVID-19 pandemic reported very high levels of burnout among ICU staff, affecting both physicians and nurses, and underscoring the psychological burden of critical care work under conditions of extreme workload and clinical pressure [47,48].

COVID-19 assignment was also identified as a major stressor due to the increased psychological and occupational demands of pandemic care. In our analysis, not being assigned to a COVID-19 unit emerged as the strongest protective factor, markedly reducing the risk of both emotional exhaustion and perceived stress. The odds of high emotional exhaustion were about 11 times lower, and the risk of high perceived stress about 2.5 times lower, among healthcare workers not assigned to COVID-19 units. In consistent with our findings, a large multinational study showed that healthcare professionals exposed to COVID-19 patients had a significantly higher risk of burnout, confirming that direct involvement in pandemic-related care contributes substantially to emotional exhaustion [49]. While large surveys in China and the United States confirmed significantly greater psychological distress and stress among frontline staff, particularly nurses and women directly involved in COVID-19 care, the U.S. data showing a 3.71 times higher risk of stress for those working in COVID-19 units compared with non-COVID units [50,51]. These findings can be attributed to the extreme working conditions imposed by the COVID-19 pandemic, such as rapid patient deterioration, elevated mortality, staff shortages, and complex ethical dilemmas, all of which exacerbated stress and burnout among healthcare professionals.

Shift work is a scheduling system that enables organizations to operate continuously by dividing labor into shifts [52]. Our results indicated that an on-call work schedule was significantly associated with higher levels of stress and burnout, including emotional exhaustion and depersonalization. This is consistent with previous evidence showing that shift work disrupts circadian rhythms, reduces opportunities for recovery and social interaction, and increases the risk of burnout, sleep disturbances, and other mental health problems [53]. Nurses working night shifts are particularly vulnerable, as fragmented workflows, strained interpersonal dynamics, and long or irregular schedules contribute to emotional stress, mood disturbances, safety risks, and adverse physiological effects [54,55]. Extended shifts of at least 12 h have also been identified as a critical factor heightening burnout levels among nurses, underscoring the impact of shift length on workplace well-being [56,57]. These findings could be attributed to dramatic changes in work organization during the COVID-19 epidemic, which resulted in higher workloads, unstable schedules, and frequent on-call tasks. Such situations disturbed rest recovery cycles, increased emotional demands, and limited possibilities for social support, all of which may have contributed to higher levels of reported stress and burnout among healthcare professionals.

Sleep disturbances were also assessed for their impact on psychological well-being. Healthcare professionals particularly nurses and physicians are highly vulnerable to such problems under the increased workplace stress of the COVID-19 pandemic [58]. In our study, sleep disturbances proved to be a major associated factor, they were highly prevalent (62.5%) and strongly associated with greater levels of perceived stress and burnout, including emotional exhaustion, depersonalization, and reduced personal accomplishment (*p* < 0.001). These results align with previous evidence. Pataka et al. reported significantly poorer sleep quality during the second wave of COVID-19, particularly among primary care staff, while Shechter et al. found that nearly all healthcare workers experienced poor sleep during the pandemic, with a considerable proportion suffering from moderate to severe insomnia and more than half meeting criteria for burnout [59]. In addition, our regression models confirmed that the absence of sleep disorders had a protective effect, lowering the odds of perceived stress by 76% and emotional exhaustion by 50%. These findings are reinforced by previous research showing that nurses experiencing moderate and high levels of occupational stress were significantly more likely to suffer from insomnia (OR = 1.53 and OR = 2.04, respectively), further highlighting the strong association between stress and sleep disturbances in clinical settings [60,61]. These findings demonstrate how sleep disturbances act not only as symptoms but also as significant contributors to stress and burnout.

Finally, the high prevalence of stress and burnout, along with their associated factors identified in this study, highlights the urgent need for interventions targeting modifiable workplace conditions. In this context, employee-centered flexible working time policies and practices contribute significantly to improving overall worker well-being [62]. Building on this, actions such as reducing on-call duties, providing psychological support, improving sleep hygiene, and rotating ICU staff may help mitigate long-term psychological consequences. These organizational strategies are particularly crucial in resource-constrained settings, where healthcare personnel operate under chronic overload.

International experiences provide valuable insights for designing sustainable and context-specific interventions. In Brazil, the COMVC-19 program was developed as an institutional initiative to promote mental health through prevention, education, and direct psychological assistance for healthcare workers [63]. In Mexico, digital platforms were widely adopted, with e-health interventions and teleconsultation services implemented to provide accessible psychological support for healthcare workers [64]. In the United States, short cognitive-behavioral therapy (CBT)-based programs have shown promising results in enhancing coping skills and reducing clinical symptoms among healthcare personnel [65]. Adapting these international experiences to the Moroccan context could guide sustainable and context-specific interventions to protect the well-being of healthcare professionals post-pandemic. Future research should also consider integrating biological markers such as salivary or hair cortisol, melatonin, or sleep actigraphy. This approach would help confront self-reported data with internal dose indicators, providing a more objective assessment of the physiological burden of stress and irregular work schedules.

This study has several limitations that should be considered when interpreting the findings. First, its cross-sectional design captures associations at a single point in time and therefore precludes any causal inference. Second, Residents were excluded from the study because their training status and working conditions differ substantially from those of permanent healthcare staff, which could introduce heterogeneity into the analysis. Similarly, healthcare workers under psychotropic medication were excluded in order to avoid potential bias in the assessment of stress and treatment effects might influence burnout, as their responses. Third, the reliance on self-reported instruments (PSS-14 and MBI) may have introduced reporting, recall, or social desirability bias. Fourth, despite the high response rate, non-response bias cannot be fully excluded, as those who declined participation may have had different levels of stress or burnout. Finally, unmeasured confounders may have influenced the observed associations despite statistical adjustment.

## 5. Conclusions

This study highlights the considerable psychological burden faced by healthcare professionals in Morocco during the COVID-19 pandemic, with more than half reporting high levels of perceived stress, emotional exhaustion, and burnout syndrome. Occupational factors such as ICU assignment, COVID-19 exposure, shift work, and sleep disorders were the strongest predictors, whereas sociodemographic characteristics played a less prominent role. These findings underscore the urgent need for institutional reforms to improve working conditions, reduce emotional demands, and strengthen psychosocial support for frontline staff. Importantly, they emphasize the necessity of establishing long-term monitoring systems and preventive interventions tailored to low- and middle-income healthcare settings, where structural and resource constraints exacerbate vulnerability. Practically, institutions should ensure systematic monitoring of stress and burnout, offer mental health support services, regulate shift schedules to avoid overload, and promote resilience-building training for healthcare staff. Future research should adopt longitudinal and multicenter approaches to better capture the evolution of stress and burnout, and to evaluate the effectiveness of targeted strategies aimed at safeguarding healthcare workers’ mental health in times of crisis.

## Figures and Tables

**Table 1:** Sociodemographic Factors and Job Characteristics of the Participants.

	*n* = 200	
Variables	Frequency (*n*)	Percentage (%)
Gender		
Female	120	60.0
Male	80	40.0
Marital Status		
Unmarried	85	42.5
Married	115	57.5
Occupation		
Nurse	146	73.0
Doctor	54	27.0
Department		
Medical department	71	35.5
Surgical department	43	21.5
COVID-Department	47	23.5
Intensive Care Unit	39	19.5
WorkShift		
Administrative schedule	77	38.5
On-call system	123	61.5
COVID Assignment		
Yes	133	66.5
No	67	33.5

**Table 2:** Distribution of Perceived Stress and Burnout Levels among Participants.

	PSS		EE		DP		PA	
	*n*	%	*n*	%	*n*	%	*n*	%
Low	30	15.0	41	20.5	81	40.5	102	51.0
Moderate	66	33.0	55	27.5	50	25.0	47	23.5
High	104	52.0	104	52.0	69	34.5	51	25.5

PSS: Perceived stress scale, EE: Emotional exhaustion, DP: Depersonalization, PA: Personal accomplishment, *n*: Number, %: Percentage.

**Table 3:** Frequency of Burnout Severity Categories.

	BO	
	*n*	%
No burnout	35	17.5
Moderate burnout	64	32.0
Burnout syndrome	101	50.5

BO: Burn out, *n*: Number, %: Percentage.

**Table 4:** Bivariate associations between sociodemographic variables and perceived stress.

		PSS			
		Low	Moderate	High	Sig
Gender	Female	12 (9.9%)	36 (29.8%)	73 (60.3%)	
	Male	18 (22.8%)	30 (38.0%)	31 (39.2%)	0.006 *
Marital Status	Unmarried	12 (14.1%)	25 (29.4%)	48 (56.5%)	
	Married	18 (15.7%)	41 (35.7%)	56 (48.7%)	0.543
Occupation	Nurse	15 (10.3%)	44 (30.1%)	87 (59.6%)	
	Doctor	15 (27.8%)	22 (40.7%)	17 (31.5%)	0.000 *
Department	Medical Department	14 (19.7%)	33 (46.5%)	24 (33.8%)	
	COVID Department	4 (8.5%)	13 (27.7%)	30 (63.8%)	
	Surgical Department	10 (23.3%)	16 (37.2%)	17 (39.5%)	
	Intensive Care Unit	2 (5.1%)	4 (10.3%)	33 (84.6%)	0.000 *
Workshift	Administrative schedule	26 (33.8%)	31 (40.3%)	20 (26.0%)	
	On-call system	4 (3.3%)	35 (28.5%)	84 (68.3%)	0.000 *
COVID Assignment	No	23 (34.3%)	23 (34.3%)	21 (31.3%)	
	Yes	7 (5.3%)	43 (32.3%)	83 (62.4%)	0.000 *
Sleep Dissorders	No	25 (33.3%)	33 (44.0%)	17 (22.7%)	
	Yes	5 (4.0%)	33 (26.4%)	87 (69.6%)	0.000 *

PSS: Perceived stress scale, *: <0.05 indicates statistical significance

**Table 5:** Relationship between sociodemographic factors and burnout dimensions.

		EE				DP				PA			
Variables		Low	Moderate	High	Sig	Low	Moderate	High	Sig	Low	Moderate	High	Sig
Gender	Female	29 (24.0%)	27 (22.3%)	65 (53.7%)		47 (38.8%)	28 (23.1%)	46 (38.0%)		68 (56.2%)	24 (19.8%)	29 (24.0%)	
	Male	12 (15.2%)	28 (35.4%)	39 (49.4%)	0.083	34 (43.0%)	22 (27.8%)	23 (29.1%)	0.421	34 (43.0%)	23 (29.1%)	22 (27.8%)	0.161
Marital Status	Unmarried	12 (14.1%)	24 (28.2%)	49 (57.6%)		29 (34.1%)	23 (27.1%)	33 (38.8%)		45 (52.9%)	20 (23.5%)	20 (23.5%)	
	Married	29 (25.2%)	31 (27.0%)	55 (47.8%)	0.144	52 (45.2%)	27 (23.5%)	36 (31.3%)	0.281	57 (49.6%)	27 (23.5%)	31 (27.0%)	0.846
Occupation	Nurse	22 (15.1%)	36 (24.7%)	88 (60.3%)		48 (32.9%)	38 (26.0%)	60 (41.1%)		85 (58.2%)	33 (22.6%)	28 (19.2%)	
	Doctor	19 (35.2%)	19 (35.2%)	16 (29.6%)	0.000 *	33 (61.1%)	12 (22.2%)	9 (16.7%)	0.281	17 (31.5%)	14 (25.9%)	23 (42.6%)	0.001 *
Department	Medical Department	19 (26.8%)	26 (36.6%)	26 (36.6%)		39 (54.9%)	13 (18.3%)	19 (26.8%)		27 (38.0%)	22 (31.0%)	22 (31.0%)	
	COVID Department	8 (17.0%)	7 (14.9%)	32 (68.1%)		13 (27.7%)	15 (31.9%)	19 (40.4%)		30 (63.8%)	9 (19.1%)	8 (17.0%)	
	Surgical Department	12 (27.9%)	13 (30.2%)	18 (41.9%)		23 (53.5%)	7 (16.3%)	13 (30.2%)		20 (46.5%)	8 (18.6%)	15 (34.9%)	
	Intensive Care Unit	2 (5.1%)	9 (23.1%)	28 (71.8%)	0.001 *	6 (15.4%)	15 (38.5%)	18 (46.2%)	0.001 *	25 (64.1%)	8 (20.5%)	6 (15.4%)	0.040 *
Workshift	Administrative schedule	34 (44.2%)	22 (28.6%)	21 (27.3%)		47 (61.0%)	17 (22.1%)	13 (16.9%)		23 (2.9%)	19 (24.7%)	35 (45.5%)	
	On-call system	7 (5.7%)	33 (26.8%)	83 (67.5%)	0.000 *	34 (27.6%)	33 (26.8%)	56 (45.5%)	0.000 *	79 (64.2%)	28 (22.8%)	16 (13.0%)	0.000 *
COVID Assignment	No	34 (50.7%)	22 (32.8%)	11 (16.4%)		44 (65.7%)	15 (22.4%)	8 (11.9%)		14 (20.9%)	16 (23.9%)	37 (55.2%)	
	Yes	7 (5.3%)	33 (24.8%)	93 (69.9%)	0.000 *	37 (27.8%)	35 (26.3%)	61 (45.9%)	0.000 *	88 (66.2%)	31 (23.3%)	14 (10.5%)	0.000 *
Sleep Dissorders	No	27 (36.0%)	29 (38.7%)	19 (25.3%)		48 (64.0%)	14 (18.7%)	13 (17.3%)		23 (30.7%)	20 (26.7%)	32 (42.7%)	
	Yes	14 (11.2%)	26 (20.8%)	85 (68.0%)	0.000 *	33 (26.4%)	36 (28.8%)	56 (44.8%)	0.000 *	79 (63.2%)	27 (21.6%)	19 (15.2%)	0.000 *

EE: Emotional Exhaustion, DP: Depersonalization, PA: Personal Accomplishment, Sig: significance, *: <0.05 indicates statistical significance

**Table 6:** Predictive factors of perceived stress among healthcare professionals: Results from ordinal logistic regression (n = 200).

Predictor	B (Estimate)	Odds Ratio (Exp (B))	95% CI for OR	*p*-Value
Occupation (Nurse vs. Doctor)	0.173	1.19	[0.57–2.48]	0.645
Department (Medical vs. ICU)	−2.038	0.13	[0.05–0.38]	<0.001
Department (Surgical vs. ICU)	−0.811	0.45	[0.14–1.42]	0.172
Department (COVID vs. ICU)	−2.059	0.13	[0.04–0.40]	<0.001
Workshift (Admin vs. On-call)	−1.137	0.32	[0.16–0.66]	0.002
COVID Assignment (No vs. Yes)	−0.952	0.39	[0.20–0.75]	0.005
Sleep Disorders (No vs. Yes)	−1.416	0.24	[0.12–0.49]	<0.001

Model fit: χ^2^ = 102.25, df = 7, *p* < 0.001; Nagelkerke R^2^ = 0.464; AIC = 175.2. OR = Odds Ratio; CI = Confidence Interval; ICU = Intensive Care Unit.

**Table 7:** Predictive factors of emotional exhaustion among healthcare professionals: Results from ordinal logistic regression (*n* = 200).

Predictor	B (Estimate)	Odds Ratio (Exp (B))	95% CI for OR	*p*-Value
Occupation (Nurse vs. Doctor)	0.248	1.28	[0.61–2.71]	0.516
Department (Medical vs. ICU)	−1.419	0.24	[0.09–0.63]	0.004
Department (Surgical vs. ICU)	−0.115	0.89	[0.30–2.62]	0.834
Department (COVID vs. ICU)	−1.419	0.24	[0.09–0.69]	0.008
Workshift (Admin vs. On-call)	−1.037	0.35	[0.17–0.73]	0.005
COVID Assignment (No vs. Yes)	−2.369	0.09	[0.05–0.19]	<0.001
Sleep Disorders (No vs. Yes)	−0.7	0.50	[0.24–1.01]	0.053

Model fit: χ^2^ = 117.75, df = 7, *p* < 0.001; Nagelkerke R^2^ = 0.511; AIC = 170.1. OR = Odds Ratio, CI = Confidence Interval; ICU = Intensive Care Unit.

## Data Availability

The data presented in this study can be obtained from the corresponding author up-on reasonable request. With regard to institutional and ethical limitations, the dataset is not publicly accessible.
